# A Renewed Focus on the Association Between Thyroid Hormones and Lipid Metabolism

**DOI:** 10.3389/fendo.2018.00511

**Published:** 2018-09-03

**Authors:** Leonidas H. Duntas, Gabriela Brenta

**Affiliations:** ^1^Unit of Endocrinology Diabetes and Metabolism, Thyroid Section, Evgenideion Hospital, University of Athens, Athens, Greece; ^2^Unit of Endocrinology and Metabolism, Thyroid Section, Dr. Cesar Milstein Hospital, Buenos Aires, Argentina

**Keywords:** cholesterol, LDL, TSH, hypothyroidism, triglyceride, thyroxine

## Abstract

Thyroid dysfunction, manifesting as either overt or subclinical hypothyroidism, negatively affects lipid metabolism: this leads to hypercholesterolemia which progressively increases the risk for cardiovascular disease and, potentially, mortality. Hypercholesterolemia in hypothyroidism is mainly due to a reduction in low-density lipoprotein (LDL) receptor activity, this accompanied by concomitant diminishing control by triiodothyronine (T3) of sterol regulatory element-binding protein 2 (SREBP-2), which modulates cholesterol biosynthesis by regulating rate-limit degrading enzyme 3-hydroxy-3-methylglutaryl-coenzyme A reductase (HMG-CoA) activity. Recently, 3,5-diiodothyronine (T2), a natural thyroid hormone derivative, was found to repress the transcription factor carbohydrate-response element-binding protein (ChREBP) and also to be involved in lipid catabolism and lipogenesis, though via a different pathway than that of T3. While thyroid hormone could therapeutically reverse the dyslipidemic profile commonly occurring in hypothyroidism, it should be borne in mind that the potency of the effects may be age-and sex-dependent. Thyroid hormone administration possibly also sustains and enhances the efficacy of hypolipidemic drugs, such as statins, ezetimibe and proprotein convertase subtilisin/kexin type 9 (PCSK9), in patients with dyslipidemia and hypothyroidism.

## Introduction

The link between thyroid diseases and serum cholesterol was firmly established following the classic article by Mason et al. on Christmas Day of 1930, shedding light on the significance of thyroid function in cholesterol metabolism (1). In 1951, Scow reported increased fat accumulation in the tissues of hypothyroid mouse models, as compared to controls, thereby corroborating the observations of von Noorden, in Vienna in 1900, that the thyroid gland plays a pivotal role in the development of “fatty disease” (2, 3). Since then, hypothyroidism has been associated with obesity and hypercholesterolemia, the extent of the latter usually being greater in primary than in secondary hypothyroidism due to the more severe condition of the primary form (4, 5).

The prevalence of hypothyroidism is 1.4–13% in patients with hyperlipidemia, indicating that thyroid failure is common and may often go undetected in these patients (6, 7). Lipid levels increase in a graded fashion as thyroid function declines, while patients with TSH values between 5.1 and 10 mIU/L have significantly higher mean total cholesterol (TC) and low-density lipoprotein-cholesterol (LDL-C) levels as compared to euthyroid subjects. In a retrospective cohort study aiming to determine the prevalence of thyroid dysfunction in 8,795 patients of various races/ethnicities, TSH was found to be high in 5.2% of the 49.5% of patients who were diagnosed with hyperlipidemia (8). Specifically, 3.5% had a TSH level of 5 to 10 mIU/L and 1.7% had a TSH level >10 mIU/L, suggesting that even subclinical hypothyroidism (SCH) might be a secondary cause of hyperlipidemia and thus be linked to coronary heart disease (CHD).

In hypothyroidism, the dyslipidemia is mainly caused by a shift to increased synthesis over degradation rate, with the elevated levels of TC, chiefly LDL-C, providing the substrate for lipid peroxidation by reactive oxygen species (ROS), this resulting in oxidative stress (9). Moreover, the synthesis and rate of catabolism of fatty acids in hypothyroidism is decreased and the lipolytic sensitivity of white fat cells is blunted (10). Crucially, the coexistence of dyslipidemia and hypothyroidism is closely linked to the development of CHD, which is the leading cause of death in most parts of the world (11). The fact that the degree of thyroid failure influences the metabolism of cholesterol has often given rise to discussions during interdisciplinary meetings as to whether the thyroid should first be treated and what is the cut-off of thyrotropin (TSH) to start treatment (12).

The development of powerful hypolipidemic drugs, such as proprotein convertase subtilisin/kexin type 9 (PCSK9), which may be optimally combined with L-thyroxine (L-T4) to treat severe forms of familial hypercholesterolemia and hypothyroidism has opened up a new field of research investigating the underlying mechanisms connecting thyroid function to lipids (13).

The aim of this review is to examine the knowledge acquired mainly over the past decade concerning the intriguing connections between thyroid hormone and lipid metabolism at the molecular level and to present a clinical assessment.

## The various mechanisms connecting thyroid hormone to lipids

Cholesterol is generated in the liver by the enzyme 3-hydroxy-3-methylglutaryl-CoA (HMG-CoA) and is transported through the circulation by lipoproteins, these being classified according to their size and density. Among the various lipoprotein subfractions, LDL-C has attracted much attention (14) due to its atherogenicity, susceptibility to oxidation and potential to predict risk for CHD. Meanwhile, also of interest is high-density lipoprotein cholesterol (HDL-C), due to its mediation of cholesterol reverse transport from the circulation to the liver and the cardiovascular protective effects that it exerts. Thyroid hormone is the main regulator of lipid metabolism by stimulating the mobilization and degradation of lipids as well as *de novo* fatty acid synthesis in the liver (15). T3 actions are mediated via modulation of gene expression and cell signaling pathways, while cholesterol synthesis is mediated by the sensing of intracellular cholesterol in the endoplasmic reticulum via sterol regulatory element binding proteins (SREBP)−1 and−2, the transcription factor that positively regulates the expression of LDL receptor (LDLR) and cholesterol synthesis (15, 16). After cleavage by specific proteases, SREBP migrates to the nucleus and acts as a transcription factor binding to the sterol regulatory element (SRE) which stimulates the transcription of the LDLR and HMG-CoA reductase genes.

Moreover, T3 is known to regulate thermogenesis and reduce body weight by stimulating brown adipose tissue (BAT) activity and increasing mitochondrial uncoupling protein 1 (Ucp1) gene transcription (17). Recently, the carbohydrate-responsive element-binding protein (ChREBP) was identified as a T3 target gene in BAT, since stimulation of ChREBP by T3 results in a 5.2-fold upregulation of Ucp1, this indicating that thyroid hormone fine-tunes hepatic lipogenesis via modulation of both SREBP-1 and ChREBP gene expression (18). Interestingly, this process is likely to be mediated through activation of thyroid hormone receptor beta (TRβ), which has been found in the liver and adipocytes (19). It is highly interesting that the metabolite of triiodothyronine (T3), 3,5-diiodo-l-thyronine (T2), although mimicking the actions of T3 on hepatic lipids, exerts an inhibitory effect on *de novo* lipogenesis that renders this compound a potential selective drug for certain metabolic conditions (15, 20). Transfection studies have shown that T2 does not act via TRβ but through an increase of hepatic nuclear sirtuin 1 (SIRT1) activity that targets peroxisome proliferator-activated receptor (PPAR)-γ coactivator (PGC-1α) and SREBP-1c; these deacetylation processes result in mitochondrial biogenesis and downregulation of lipogenic genes (21). T3 exerts its effects through both mechanisms: genomic, consisting in linking T3 to nuclear receptors that bind responsive elements in the promoter of target genes, and non-genomic, by αvb3 receptor-mediated MAPK/ERK and PI3K/Akt/mTOR-C1 activation (22). T2, unlike T3, as was shown in HepG2-cells, determines the block of proteolytic cleavage of SREBP-1 without affecting its expression at the transcriptional or translational level (23). T2 concurrently activates MAPKs ERK and p38, of the Akt and PKC-δ pathways, resulting in apoptosis of HepG2 cells. In animals fed a high-fat diet (HFD), T2 prevented both adiposity and body weight gain by increasing lipid mobilization and hepatic beta-oxidation. On the other hand, by increasing lipid catabolism, T3 enhances hepatic lipid accumulation while also simultaneously elevating lipogenesis. The above observations highlight the different molecular mechanisms of T3 and T2 activity in their inhibition of hepatic lipid accumulation (23).

**Figure 1:**
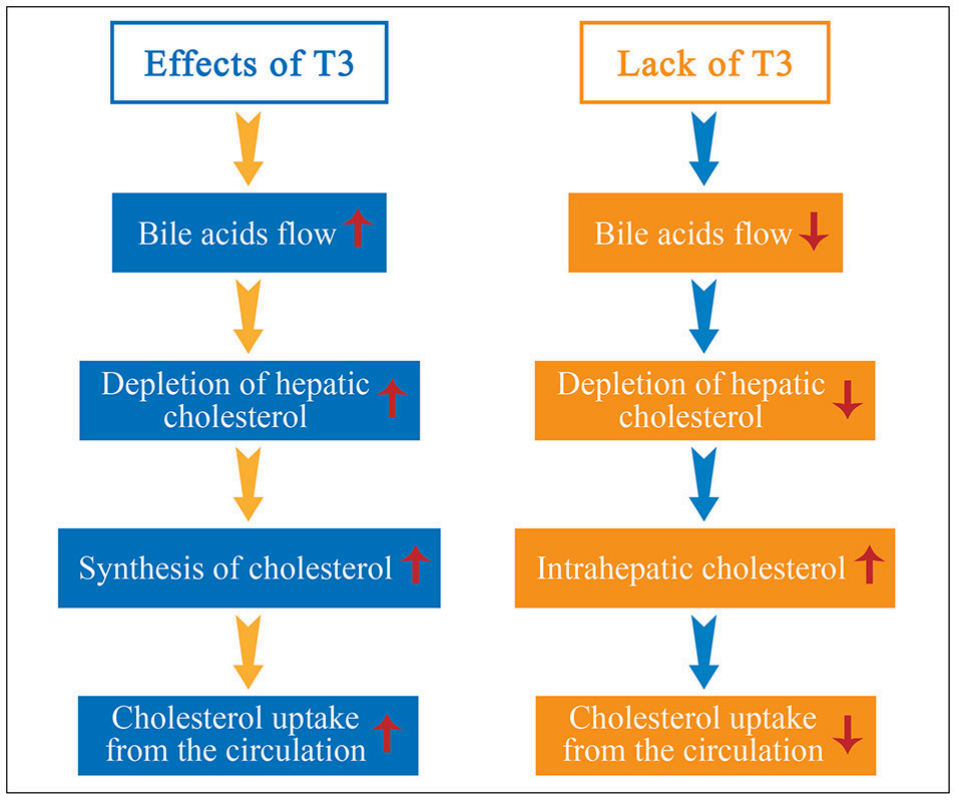
Thyroid hormone (TH) increases the synthesis of cholesterol and bile acids (BA) flow, resulting in depletion of hepatic cholesterol and enhancement of cholesterol uptake from the circulation to the liver. By contrast, in hypothyroidism, diminution of TH results in slowing of BA flow, marked diminution in the rate of cholesterol secretion into the bile, increase of intrahepatic cholesterol despite the decrease in cholesterol biosynthesis, and decrease of hepatic uptake of cholesterol from the circulation.

Cholesterol is converted to cholesteryl esters by lecithin-cholesterol acyltransferase (LCAT) and is transferred from HDL to apolipoprotein B (apoB)-containing lipoproteins by cholesterol ester transfer protein (CETP) (24). CETP has a pivotal role in the reverse cholesterol transport mechanism, a bilateral key process protecting vessel walls against atherosclerosis. The enzyme hepatic lipase (HL) regulates the hydrolysis of HDL2 to HDL3, whereas the lipoprotein lipase (LPL) catabolizes serum triglycerides and transports free cholesterol to HDL (5, 24). The activity of CETP, HL and LPL is regulated by thyroid hormone, this strongly pointing to its crucial influence in cholesterol metabolism. Moreover, thyroid hormone increases the flow of bile acids (BA) causing the depletion of intrahepatic cholesterol and the enhancement of cholesterol synthesis in the liver and of hepatic uptake of cholesterol from the circulation, thus maintaining the balance of hepatic cholesterol (25). By contrast, in hypothyroidism, a decline of TH levels results in slowing of BA flow, marked diminution in the rate of cholesterol secretion into the bile, increase of intrahepatic cholesterol despite the decrease in cholesterol biosynthesis and decrease of hepatic uptake of cholesterol from the circulation (26) (Figure 1). In parallel with lowering of cholesterol excretion there is a rise in LDL-C by a factor of ~3, the latter being due to decreased LDL-receptor (LDLR) activity accompanied by suppression of the uptake of LDL by the LDLR receptor in the liver. Indeed, LDLR mRNA levels decrease by nearly 50%, indicating that LDLR is regulated at the mRNA level (27). The result appears to be reduced catabolism and turnover, which would account for the presence of dyslipidemia in hypothyroidism.

The effects of thyroid hormone on the various fractions of lipids are presented in Table 1.

**Table 1:** The effects of thyroid hormone on the various fractions of lipids.

	**TC**	**LDL-C**	**HDL-C**	**Triglycerides**	**ApoB**	**Lp(α)**
LT4	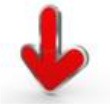	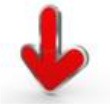	*n* ( 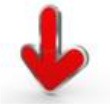 )	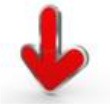	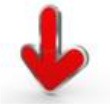	*n* ( 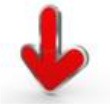 )^*^

TC, Total Cholesterol; LDL-C, Low Density Lipoprotein Cholesterol;

HDL-C, High Density Lipoprotein Cholesterol;

ApoB, Apolipoprotein B; LP(α), Lipoprotein (α).

* *Depending on lipoprotein (α) molecular weight*.

Recently, a cross-sectional investigation conducted in 250 patients with Graves' orbitopathy (GO) revealed that those with disease lasting <44 months had higher serum TC and LDL-C levels, an association confirmed by the results of the Clinical Activity Score (CAS) in untreated patients (28). The findings suggest that cholesterol levels above 191 mg/dl could represent a novel independent risk factor for GO. These results have more recently been corroborated in 86 consecutive patients with new-onset GD (29). Serum levels of TC and LDL-C were significantly higher in patients with GO (211.6 ± 44.0 and 135.3 ± 41.3 mg/dL, respectively) than in those without GO (176.0 ± 27.2 and 106.6 ± 23.9 mg/dL, respectively). No relationship between GO severity and activity and cholesterol was found. The mechanisms are not clear. The fact that the associations between TC and LDL-C were observed in patients with GO of recent onset points to the potential involvement of autoimmune process *per se* and to the possibility that TC is involved in the induction of oxidative stress which plays an important role in the pathogenesis of GO. Meanwhile, the recent finding that TSH-β mRNA was positively associated with serum TC and LDL-C and, moreover, that it was detected in adipose tissue (30), might partially substantiate these results and hypotheses.

## Triglyceride remnants, residual CVD risk, and thyroid hormone

In spite of the fact that LDL–C is the primary lipid target for cardiovascular disease (CVD) prevention, other lipid measures should be undertaken to assess individuals with well controlled LDL-C levels who are still exposed to high residual risk of CVD which is the term used to define the CVD risk that remains despite intensive statin treatment (31). Residual CVD risk is mainly determined by hypertriglyceridemia, elevated small dense LDL particles, reduced HDL-C and HDL particle numbers, increased triglyceride (TG)–rich lipoproteins or remnant lipoproteins (RLPs) and postprandial hyperlipidemia, also known collectively as the atherogenic dyslipidemia complex (32). Markers of residual risk include a number of biochemical parameters, such as non-HDL-C, that reflect the cholesterol content of RLPs (33), as previously shown in diabetic patients (34). RLPs increase intimal cholesterol deposition and activate several proinflammatory, proapoptotic and procoagulant pathways (35). Medium-sized TG-rich RLPs, present in mild to moderate hypertriglyceridemia, can enter the arterial wall and cause atherosclerosis (36). Indeed, clinical conditions related to high CVD risk, such as obesity, metabolic syndrome (MetS) and type 2 diabetes mellitus (T2DM), are associated with RLPs accumulation and elevated inflammatory markers which remain despite statin treatment (37).

In this regard, although overt hypothyroidism (OH) has been traditionally associated with increased levels of cholesterol, an elevation of TG and RLPs levels both in overt and SCH patients have also been reported. Furthermore, hypertriglyceridemia, one of the main components of the MetS, was found to be associated with higher TSH values in several populations that included patients with MetS. In fact, in the MJ Health Screening database with 94,434 participants, patients with MetS were at a 21% excess risk of developing SCH, while after 4.2 years, when individual components were analyzed, an increased risk of SCH was associated with high serum triglycerides (38).

It is of note that subtle changes in TH have a significant opposite effect on plasma VLDL-TG concentration, which was found to be higher in SCH, this due to greater secretion of large TG-rich VLDL particles from the liver, and lower in subclinical hyperthyroidism than in euthyroid subjects (39). The action of thyroid hormones on triglyceride metabolism includes both *de novo* lipogenesis via the transcription of several key lipogenic genes (40) and increased hydrolysis: the latter is regulated by its actions on LPL (41) and HL activity, the enzyme that converts intermediate-density lipoprotein (IDL) to LDL (42, 43). With regard to LPL, it has been reported that T3 can stimulate its activity both by upregulating apolipoprotein AV (ApoAV) (44) and by decreasing its inhibitor angiopoietin-like proteins 3 (ANGPTL3) (45).

Thus, one explanation for the increase in plasma TG levels in hypothyroidism is the decreased clearance of TG-rich lipoproteins through lower enzymatic activity. In this context, a decline of HL activity in SCH was reported in association with impaired chemical composition of isolated LDL particles due to TG enrichment in middle-aged women with SCH (46). HL activity was subsequently found to be lower in the presence of VLDL remnants/IDL, which are identified via an electrophoretic qualitative method in the fasting plasma of patients with SCH (47). By use of an immunoaffinity-chromatography method for evaluating the cholesterol concentration of RLPs known as remnant-like particle cholesterol (RLP-C), increased RLP-C levels in SCH women were conclusively confirmed (48). Moreover, among the latter group of patients, RLP-C levels decreased upon 6 months of L-T4 treatment together with an increase in HL activity (48). As immunoaffinity-chromatography is not routinely performed, the presence of RLPs can also be estimated by assessing non-HDL-C or non-fasting remnant cholesterol (non-fasting TC minus HDL-C minus LDL-C) (49). It is of note that there have been previous reports of a reduction of TG-rich remnants in the serum of patients with thyroid hormone deficiency following L-T4 treatment (50) and of the presence of postprandial hyperlipidemia in hypothyroidism (51).

Notwithstanding that hypertriglyceridemia in the setting of hypothyroidism develops as a result of impaired removal of endogenous TG (52), and increased hepatic production of triglycerides (53). Gjedde et al. (54) have described an overproduction by the liver of TG-rich particles in OH patients in the presence of unaltered rates of lipolysis. The normal lipolysis and high TG concentrations indicates increased TG synthesis. Similarly, plasma kinetics of an artificial TG-rich emulsion labeled with radioactive TG and cholesteryl esters was unaffected in SCH by comparison with euthyroid individuals, suggesting that TG-rich lipoprotein metabolism is unimpaired in SCH and increased levels of these atherogenic lipoproteins are mainly due to overproduction by the liver (55). In this study, TG and phospholipid transfer to HDL was indeed seen to be significantly lower in SCH subjects than in controls. In a further study, VLDL-TG and VLDL-apolipoprotein B-100 (apoB-100) kinetics were assessed in SCH patients (55). A larger secretion of large TG-rich very low-density lipoprotein (VLDL) particles from the liver with consequent higher plasma VLDL-TG concentration was detected in SCH subjects.

Another possible way to explore TG metabolism is by examining postprandial hyperlipidemia, a predictor of atherogenesis (56). By means of an oral lipid tolerance test, Tanaci et al. (52) found postprandial hyperlipidemia (an increase of TG levels by 80% or more) equally more prevalent in OH and SCH than in euthyroid individuals. Moreover, patients with a TSH level higher than 5 mIU/L had a 7-fold increased risk of postprandial hyperlipidemia, indicating that postprandial TG metabolism is affected in hypothyroidism. Arikan et al. (57) also compared euthyroid, OH and SCH patients and detected higher TG levels 8 h postprandially in hypothyroid patients than in controls. In a more recent study, apolipoprotein B48 (Apo B48), a marker of intestinally derived lipoprotein, was found to be higher in overt and SCH compared to euthyroid patients (58), though the effect of SCH was milder than that of OH. On the other hand, hypothyroidism was not observed to be associated with chylomicronemia (non-fasting TG above 177 mg/dL) in 108,711 individuals from the Copenhagen General Population Study in whom obesity and T2DM were the main risk factors (59). These results are at odds with an epidemiological study in which subjects with hypertriglyceridemia had a higher risk for SCH (60). When TG levels were higher than 150 mg/dL, this risk increased ~2.0-fold in men and 1.4-fold in women.

In summary, although the atherogenic role of LDL-C is well known and hypercholesterolemia is the most frequent lipoprotein alteration in hypothyroidism, it should be noted that higher TG-enriched lipoprotein levels are also present in the serum of hypothyroid patients. These RLPs in hypothyroidism arise from decreased lipolysis of lipoproteins due to lower enzymatic activity and possibly also through increased production by the intestine and the liver. Since RLPs have been recognized as highly atherogenic, the relevance of their presence in the serum of hypothyroid patients without replacement therapy is a fertile subject for future research.

## Age, lipid metabolism, and the thyroid

While age is an established risk factor for dyslipidemia and thus for CVD, the increased proportion of cardiac events in later life may also be attributed to the presence of other comorbidities (61). Nevertheless, despite the lack of pertinent data and the risk of adverse effects from lipid-modifying therapy, the latter approach is generally deemed worthwhile in elderly patients (62). In these subjects, it is particularly important to rule out secondary dyslipidemia, such as hypothyroidism, to avoid unnecessary statin treatment in polymedicated individuals. Notably, it has been reported that hypothyroidism frequently occurs in dyslipidemic patients (63, 64) and several guidelines (6, 65, 66) recommend case-finding with TSH determination in such a setting. However, given that slightly higher TSH values are considered physiological as age advances, care has to be taken to distinguish between the two conditions, i.e., physiologic or pathologic TSH elevation, especially when replacement with L-T4 is being considered (67). To make this decision, it is helpful to check for the presence of dyslipidemia, while also taking into account the levels of TSH elevation together with positive thyroid peroxidase antibody (TPOAB) titers.

Several studies have demonstrated that lower thyroid function can be a risk factor for a worse lipid profile in elderly patients. In a previous cross-sectional study of 2,799 individuals aged 70–79 years, TSH > 5.5 mIU/L was associated with a 9 mg/dL higher cholesterol level (68). Furthermore, in another cross-sectional study including 30,656 participants, lipid values even within the normal TSH range were directly associated with TSH values in men above 50 years of age (69). For women, associations were statistically significant in all age groups except for HDL cholesterol in women below 50 years of age. However, due to the nature of this cross-sectional study, it is not clear whether the association between higher lipid values and TSH, within the euthyroid range, is due to high TSH values *per se* or to mildly decreased thyroid function.

Elderly patients appear to be at greater risk of developing MetS and the resultant hypothyroidism could contribute to the increased prevalence. Studies were thus conducted to examine whether the prevalence and incidence of MetS are heightened due to augmented TSH levels. Once again, both, SCH and MetS were observed to be linked to increased risk of CHD. Meanwhile, the association between thyroid function and the prevalence and incidence of MetS in older patients were determined by analyzing the data from the Health, Aging and Body Composition Study, a prospective cohort of 3,075 community-dwelling US adults (70). It was shown that SCH with a TSH > 10 mIU/l was significantly associated with increased prevalence but not incidence of MetS (70). In the Longitudinal Aging Study Amsterdam, recruiting 1,187 participants (590 men and 597 women) between the ages of 65 and 88 years, it was observed that subjects with a serum TSH level above 2.28 mIU/L [odds ratio (OR) = 1.68; 95% confidence interval (CI) 1.19–2.37] had a significantly increased prevalence of MetS compared with subjects with serum TSH below 1.04 mIU/L (71). It is therefore evident that increasing TSH is likely to be associated with increasing prevalence of MetS.

The contribution of hypothyroidism to age-related dyslipidemia has been addressed by Tognini et al. who stratified 2,308 patients according to age and TSH values (72). In this study, those patients with higher TSH levels showed a continuous worsening of lipid profile with age, which was noted even in subjects just over the age of 65. More specifically, the above study reported that in patients older than 65 years, both serum LDL-C and LDL/HDL-C values significantly increased further in those with TSH between 3.6 and 10 mIU/L, while in patients with TSH > 10 mIU/L, TC levels increased as well. The main finding of this study is that hypothyroidism, both SCH and OH, could aggravate the rise of lipids with age; thus, age proved to be the most influential factor as regards serum TC levels, TSH following in importance.

In the same line of research, a subsequent study assessed the role of SCH in lipid profiles in elderly subjects and compared it with younger individuals. The analyzed population consisted of 17,046 middle-aged and elderly patients derived from the REACTION study conducted across China from 2011 to 2012 (73). After adjustment for multiple factors, it was found that each 1 mU/L increase in TSH was related to an increase of TC and LDL-C as age increased. Thus, the effect of TSH proved to be stronger on cholesterol levels in moderately old subjects (60–69 years) than in younger subjects (40–49 years). Furthermore, SCH, categorized into mild and severe (TSH <10 or >10 mIU/L) in moderately old subjects, increased the prevalence of hypercholesterolemia (≥240 mg/dL) ~1.50- and 2.27-fold, respectively, when compared with younger individuals (73). Conversely, in disagreement with previous observations (72), the study showed that the plateau in lipid levels commonly observed with advanced age was still present despite TSH elevation.

More questions arise when one looks at lipoprotein alterations associated with SCH in the oldest old segment of the population, albeit very limited data regarding lipid metabolism and thyroid function are available for this age group. The concept of reverse physiopathology in which higher TSH values are deemed desirable for increased survival seems to apply in this scenario (74).

In brief, it has been postulated that higher TSH values in the elderly are a physiological adaptation of the aging process. Hence, the association between mild thyroid failure and lipid profile in the elderly is still to be elucidated. The fact remains, nevertheless, that TSH levels above the reference range among older patients, and particularly those with levels over 10 mIU/L, have proven to be associated with higher cholesterol levels than what is observed in younger individuals.

## Treatment with L-T4: why, who, and how

**Figure 2:**
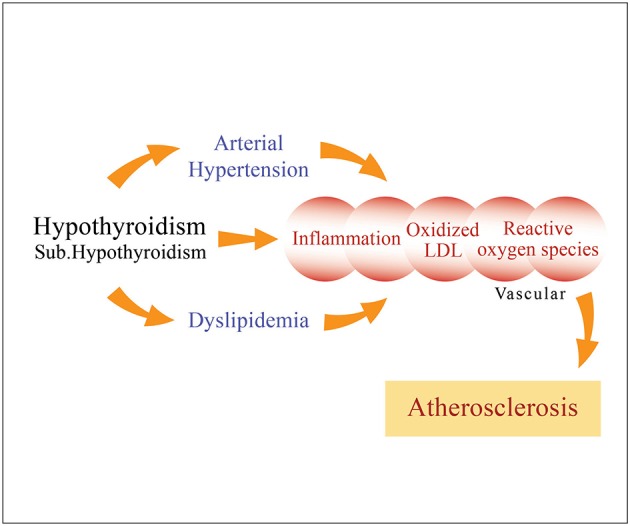
Long-term overt hypothyroidism, as well as subclinical hypothyroidism to a lesser degree, when left untreated cause dyslipidemia and arterial hypertension (diastolic), as well as inflammation characterized by oxidative stress and generation of reactive oxygen species, which may induce endothelial dysfunction, thereby promoting atherosclerosis.

Dyslipidemia, including secondary dyslipidemia that commonly arises in hypothyroidism, is one of the main risk factors for the development of atherosclerosis, the latter arising through changes in lipid profile, arterial hypertension, inflammation and/or oxidative stress which contribute to endothelial dysfunction (75) (Figure 2). Lipid status worsens, along with TSH levels. Therefore, TSH values can be considered a good predictor of cardiovascular disease, notably when its levels are above 10 mIU/L (75). In particular, a TSH above 2.5 mIU/L in women of childbearing age may induce oxidative damage to membrane lipids and unfavorably alter the lipid profile, suggesting that TSH levels in this population should preferably be maintained below 2.5 mIU/L (76).

The influence of SCH on lipids is proportional to the degree of TSH elevation and becomes increasingly evident as SCH progresses to OH, this process additionally precipitating any predisposition the patient may have to atherosclerosis (9). Of note, given that progression of the disease is accompanied by significantly decreased nitrite and nitrate levels, nitric oxide (NO) levels could be a reliable biomarker for cardiovascular risk in SCH (77). Recently, a retrospective analysis of 82 thyroidectomized patients with differentiated thyroid cancer showed that following stimulation by recombinant human TSH (rhTSH), serum T3 concentrations decreased (from 1.91 to 1.81 nmol/L; *p* < 0.001), while T4, fT4, and rT3 remained unchanged (78). Thus, these results may suggest that TSH has a direct effect on peripheral TH metabolism by decreasing T3 in patients receiving L-T4 and that the decline in T3 may cause the dyslipidemic profile. Moreover, after rhTSH stimulation, both apoB-100 and TG statistically significantly increased from 0.90 to 0.92 g/L (*p* = 0.03) and from 1.98 to 2.50 mmol/L (*P* < 0.001), respectively, while HDL-C decreased from 0.98 to 0.81 mmol/L (*p* < 0.001). It is of note that the decreasing effect of L-T4 was more pronounced in short-term (<6 months) rather than in long-term (>6 months) studies.

The efficacy of L-T4 replacement to normalize the lipid profile of hypothyroid patients is related to the degree of disease, being more evident in OH. Though there is no consensus regarding the lipid-lowering effect of replacement treatment with L-T4 in patients with SCH, a recent search in PubMed, the Cochrane Library, ClinicalTrials.gov and EMBASE, in order to compare substitution to placebo treatment or observation, revealed clear benefits of L-T4 administration for reducing TC and LDL-C in SCH patients with TSH < 10 mIU/L (79).

In a meta-analysis of 13 studies with 247 patients aiming to estimate the expected decline in serum lipid concentrations following treatment with LT4, the mean diminishment in serum TC concentration wa-0.20 mmol/L (−7.9 mg/ dL), with a 95% confidence interval of −0.09 to −0.34 while the decrease in serum LDL-C concentration was −0.26 mmol/L (−10 mg/dL), with a 95% confidence interval of −0.12 to −0.41 (80). The results show that LT4 therapy in SCH patients lowers TC and LDL-C, the high levels of reduction possibly being attributable to higher pretreatment TC levels and suboptimal LT4 doses.

Currently, the guidelines recommend against treatment in the elderly in those cases where serum TSH concentrations are between the upper normal limit of the reference range and 10 mIU/L (81). These recommendations are supported by a recent case control study evaluating the association between LT4 therapy and mortality in individuals 65 years or older with SCH and TSH values <10 mIU/L (82). On a multivariate analysis treatment with LT4 was associated with significantly increased mortality (HR = 1.19 CI 1.03–1.38) in patients 65 years or older with SCH and TSH <10.

However, it is suggested that L-T4 treatment may be considered in patients between 45 and 65 years, and particularly in subjects with comorbidities like dyslipidemia, arterial hypertension and/or insulin resistance.

Moreover, a study enlisting 100 patients, mean age 53.8 yr and mean TSH 6.6 mIU/L, who were treated for 12 weeks with L-T4 100 mcg daily, demonstrated that L-T4 therapy for SCH appreciably lowers cardiovascular risk factors while concurrently often alleviating symptoms of tiredness (83). This same treatment succeeded in lowering TC from 231 to 220 mg/dl and LDL-C from 143 to 131 mg/dl. Of particular note is the fact that the FT4 increase was the most significant variable predicting a decrease in TC.

In this connection, serum paraoxonase-1 (PON-1) activity (arylesterase activity), which may attenuate oxidative stress and thus protect against CVD, was estimated in 2,206 euthyroid individuals, to assess a possible relationship between serum PON-1 activity and TSH and TH (84). Since serum PON-1 activity was inversely associated with serum FT4 levels, other mechanisms could explain the previously reported lipid oxidation reported in hypothyroid patients (84).

It is noteworthy that in an LDLR knock-out mice study, TH reduced circulating TC and VLDL-C levels by about 70% (85). Circulating values of both apoB, apolipoprotein B (apo)B48 and apoB100 were significantly decreased, indicating that TH may reduce apoB lipoproteins via a non-LDLR pathway.

The effects of overt hypothyroidism and SCH on lipids concentrations are presented in Table 2.

**Table 2:** The impact of overt (OH) and subclinical hypothyroidism (SCH) on serum lipids concentration.

	**TC**	**LDL-C**	**HDL-C**	**Triglycerides**	**ApoB**
OH	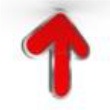	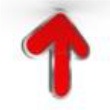	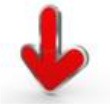	*n* ( 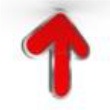 )	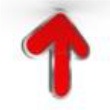
SCH TSH <10 mU/L	*n* ( 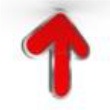 )	*n* ( 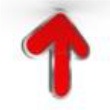 )^*^	*n* ( 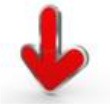 )	*n*	*n*
SCH TSH>10 mU/L	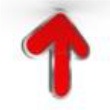	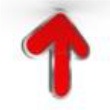	*n* ( 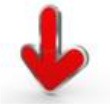 )	*n*	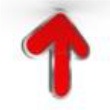

TC, Total Cholesterol;

LDL-C, Low Density Lipoproteins Cholesterol; HDL-C, High Density Lipoproteins Cholesterol; ApoB, Apolipoprotein B;

LP(α), Lipoprotein (α).

* Depending on age (<60 y or >60 y) and gender as women are more affected.

*Total and LDL-C and triglycerides increased with increasing TSH, while HDL-C decreased across the entire reference range, with no indication of a threshold effect and presumably due to gradually increased activity of CETP*.

## Thyromimetics and lipids

The different tissue specificity of TH receptors (TRs) causes variable TH effects. Thus, TRα-1 is present in skeletal and cardiac muscle, TRβ-1 is predominant in liver, kidney, and brain, TRα-2 is most prevalent in brain and testis, whereas TRβ-2 is present in the anterior pituitary gland, hypothalamus, and cochlea. TH has been shown to be effective in dyslipidemia, via its lipid-lowering action in the liver, and also in obesity. This differential pattern of TRs distribution stimulated the search for compounds with tissue-specific action on TR, leading to the development of compounds like GC1 and KB141 which selectively act on the β1 isoform of TR (86). The selective TRβ modulator GC-1 enhances several steps in reverse cholesterol transport and reduces serum cholesterol independently of LDL-R. In a study aiming to determine whether GC-1 reduces atherosclerosis and the mechanisms that are involved, apoE-deficient mice were fed an atherogenic diet ± GC-1 (87). GC-1 reduced cholesteryl esters in the aorta after 20 weeks. More recently, eprotirome, a liver-selective thyroid hormone receptor agonist, was developed and was shown to be effective in treating dyslipidemia, this due to the lipid-lowering action of TH in the liver, as well as in the treatment of obesity.

In another recent study, 236 patients were enrolled: 80 were randomly allocated to receive placebo, 79 to receive 50 μg eprotirome, and 77 to receive 100 μg eprotirome (88). There was an increase of 9% (95% CI-2 to 20) in mean LDL cholesterol concentrations in the placebo group, whereas in the 50 μg eprotirome group the concentrations dropped by 12% (−28 to 4%; *p* = 0.0677 vs. placebo) and in the 100 μg eprotirome group they fell by 22% (−32 to −13%; *p* = 0.0045 vs. placebo).

## Co-treatment with statins, ezetimibe, and proprotein convertase subtilisin/kexin type 9

Though statins are first-line treatment in the majority of patients with dyslipidemia, it must be acknowledged that they often cannot reverse the CVD risk and, moreover, that adverse effects frequently occur, compromising patient compliance (89). The side effects ranging from clinically benign myalgia to rare but life-threatening rhabdomyolysis, are likely to be dose dependent. Hence, clinicians should take into consideration a series of factors such as DM, vitamin D deficiency that potentially increase this risk. Also potentially dangerous is the coexistence of statin therapy with undiagnosed hypothyroidism, since this may elevate the risk of myopathy and even rhabdomyolysis (90). It was recently shown in a multivariate model analyzing 59,597 statin-users and non-users, that both OH and SCH were associated with an increased risk for T2DM (DM) (RR 1.53 [95% CI 1.31–1.79] and 1.75 [1.40–2.18], respectively) (91). However, OH elevated the risk for T2DM independently of use or non-use of statins, while SCH -associated risk for T2DM was prominent only upon statin use. More generally speaking, the above data emphasize the importance of identifying and treating hypothyroid patients as a means of lowering the risk for T2DM and CVD.

Another argument in favor of considering the possible presence of hypothyroidism during statin treatment is that untreated hypothyroid patients on statins rarely reach their lipid goals, the effects of atorvastatin having been demonstrated to be considerably reduced in hypothyroid patients as compared with euthyroid individuals (92). Moreover, in an animal study where LDLR expression was abolished, L-T4 still reduced lipid values. It was shown that liver production of apoB was markedly reduced by thyroid hormones and thyromimetics, thus opening up parallel avenues for statin-resistant patients (93).

Furthermore, statin therapy, as shown in a meta-analysis, produces a significant increase in plasma proprotein convertase subtilisin/kexin type 9 (PCSK9) concentrations (94). PCSK9, a serine protease, is significantly involved in the regulation of LDLR expression and apoB cholesterol metabolism. Hepatic PCSK9 protein expression, activity and secretion have been shown to affect cholesterol homeostasis and to increase LDLR degradation (95). Both currently available PCSK9 inhibitors, alirocumab and evolocumab, bind to PCSK9 preventing its binding to the LDLR: this results in continuous LDLR recycling at the hepatic cell surface and clearing of LDL-C particles, causing a decrease in plasma LDL-C concentration (95). It has been proposed that TH, by stimulating LDLR activity and clearance of LDL-C via PCSK9, may support this mechanism (13). Also of interest is the fact that the correlation of circulating PCSK9 levels with thyroid function, even in the normal range, may be blunted by obesity (96).

As a final note, treatment with ezetimibe, a selective inhibitor of Niemann-Pick C1-Like 1 (NPC1L1), an apical membrane cholesterol transporter of enterocytes, reduces intestinal cholesterol absorption (97, 98). Identification and characterization of NPC1L1 has established this protein as a critical intestinal sterol transporter that regulates whole body cholesterol metabolism (99). Moreover, ezetimibe potently inhibits the uptake and absorption of biliary and dietary cholesterol from the small intestine without affecting the absorption of fat-soluble vitamins, triglycerides, or bile acids.

Ezetimibe treatment also increases extrahepatic reverse cholesterol transport. Notably, in patients with sitosterolemia, ezetimibe, while reducing TC intestinal absorption, simultaneously decreases circulating 5α-stanol levels. Therefore, by thus lowering circulating 5α-stanols while increasing FT3/FT4, presumably by enhancing conversion of T4 to T3, it has the potential to improve thyroid hormone status (100). It is accordingly suggested that ezetimibe, together with statins, be combined with thyroxine, thereby both ameliorating thyroid function and reducing TC and LDL-C by actively inhibiting the synthesis and intestinal absorption of TC (13).

## Author contributions

All authors listed have made a substantial, direct and intellectual contribution to the work, and approved it for publication.

### Conflict of interest statement

The authors declare that the research was conducted in the absence of any commercial or financial relationships that could be construed as a potential conflict of interest.
